# Tele-rehabilitation for people with dementia in the COVID-19 pandemic: A case-study

**DOI:** 10.1192/j.eurpsy.2021.927

**Published:** 2021-08-13

**Authors:** C. Di Lorito, C. Duff, C. Rogers, J. Tuxworth, J. Bell, R. Fothergill, L. Wilkinson, A. Bosco, L. Howe, R. O’Brien, M. Godfrey, M. Dunlop, V. Van Der Wardt, V. Booth, P. Logan, R. Harwood

**Affiliations:** 1 Division Of Rehabilitation, Ageing And Wellbeing, University of Nottingham, Nottingham, United Kingdom; 2 N/a, Lincolnshire partnership NHS foundation Trust, Lincoln, United Kingdom; 3 N/a, University of Marburg, Marburg, Germany; 4 Division Of Social Sciences, University of Nottingham, Nottingham, United Kingdom

**Keywords:** Physical Activity, Tele-rehabilitation, dementia, COVID-19

## Abstract

**Introduction:**

The Promoting Activity, Independence and Stability in Early Dementia (PrAISED) is delivering an exercise programme for people with dementia. The Lincolnshire partnership NHS foundation Trust successfully delivered PrAISED through a video-calling platform during the COVID-19 pandemic.

**Objectives:**

This qualitative case-study identified participants that video delivery worked for, and highlighted its benefits and challenges.

**Methods:**

Interviews were conducted with participants with dementia, caregivers and therapists, and analysed through thematic analysis.

**Results:**

Video delivery worked best when participants had a supporting carer, when therapists showed enthusiasm and had an established rapport with the client. Benefits included time-efficiency of sessions, enhancing participants’ motivation, caregivers’ dementia awareness and therapists’ creativity. Limitations included users’ poor IT skills and resources.

**Conclusions:**

The COVID-19 pandemic required innovative ways of delivering rehabilitation. This study supports that people with dementia can use tele rehab, but success is reliant on having a caregiver and an enthusiastic and known therapist.

